# The stress-responsive cytotoxic effect of diesel exhaust particles on lymphatic endothelial cells

**DOI:** 10.1038/s41598-024-61255-4

**Published:** 2024-05-07

**Authors:** Yu Sakurai, Eiki Oba, Akiko Honda, Hiroki Tanaka, Hirohisa Takano, Hidetaka Akita

**Affiliations:** 1 Laboratory of DDS Design and Drug Disposition, Graduate School of Pharmaceutical Sciences, Tohoku University, Sendai, 980-8578 Japan; 2 Graduate School of Engineering, Kyoto University, Kyoto, 615-8530 Japan; 3 Institute for International Academic Research, Kyoto University of Advanced Science, Kyoto, 621-8555 Japan; 4 Graduate School of Global Environmental Studies, Kyoto University, Kyoto, 615-8530 Japan

**Keywords:** Lymphatic endothelial cells, Diesel exhaust particles, Cell death, Reactive oxygen species, Cell biology, Molecular biology, Environmental sciences

## Abstract

Diesel exhaust particles (DEPs) are very small (typically < 0.2 μm) fragments that have become major air pollutants. DEPs are comprised of a carbonaceous core surrounded by organic compounds such as polycyclic aromatic hydrocarbons (PAHs) and nitro-PAHs. Inhaled DEPs reach the deepest sites in the respiratory system where they could induce respiratory/cardiovascular dysfunction. Additionally, a previous study has revealed that a portion of inhaled DEPs often activate immune cells and subsequently induce somatic inflammation. Moreover, DEPs are known to localize in lymph nodes. Therefore, in this study we explored the effect of DEPs on the lymphatic endothelial cells (LECs) that are a constituent of the walls of lymph nodes. DEP exposure induced cell death in a reactive oxygen species (ROS)-dependent manner. Following exposure to DEPs, next-generation sequence (NGS) analysis identified an upregulation of the integrated stress response (ISR) pathway and cell death cascades. Both the soluble and insoluble components of DEPs generated intracellular ROS. Three-dimensional Raman imaging revealed that DEPs are taken up by LECs, which suggests internalized DEP cores produce ROS, as well as soluble DEP components. However, significant cell death pathways such as apoptosis, necroptosis, ferroptosis, pyroptosis, and parthanatos seem unlikely to be involved in DEP-induced cell death in LECs. This study clarifies how DEPs invading the body might affect the lymphatic system through the induction of cell death in LECs.

## Introduction

Air pollution is one of the most severe environmental pollutants and is harmful to humanity^1^. The World Health Organization (WHO) defines air pollution as “contamination of the indoor or outdoor environment by any chemical, physical or biological agent that modifies the natural characteristics of the atmosphere and has issued warnings of the attendant health hazards caused by air pollution^2^. For decades epidemiological studies have indicated a strong, positive correlation between air pollution and death caused mainly by lung cancer and cardiopulmonary diseases^3–5^. Such health hazards due to air pollution are particularly pronounced in China, India and other emerging economies where air pollutant emissions are increasing commensurate with rapid economic growth^6^. The primary air pollutant is particulate matter (PM) with an aerodynamic diameter of < 2.5 μm, which is referred to as PM2.5. PM is an airborne complex of particulates, both organic and inorganic, that are generated from the operation of motor vehicles, boilers, incinerators, ships, and aircraft^7^.

A significant component of PM is diesel exhaust particles (DEPs), which are emitted by the combustion of light oil in diesel engines. Although the characteristics of DEPs vary depending on the fuel mixture and operating conditions, DEPs generally consist of a solid carbon core with a large surface area and multiple shell components such as polycyclic aromatic hydrocarbons (PAHs), nitro-PAHs, and small amounts of organic compounds, sulfates, and metals^8–10^. In human cells, these components generate reactive oxygen species (ROS) and consequently provoke oxidative stress^11^. Because DEPs are fine particles (typically < 2.5 μm), inhaled DEPs reach the deepest areas in the lungs, and portions of the DEPs can be distributed to various organs via blood circulation^9^. Exposure to DEPs induces the production of cytokines^12^, pulmonary inflammation^13^, cardiovascular diseases^14^, mutagenesis^15^, and neuroinflammation^16^.

A plausible cause for these adverse symptoms is DEP-mediated inflammation and/or cytotoxicity. Airway epithelial cells are the first cells that contact inhaled DEPs, and they release inflammatory cytokines such as interleukin (IL)-8, IL-6, and granulocyte macrophage colony-stimulating factor (GM-CSF)^17,18^. When exposed to DEPs, epithelial cell viability is compromised in the respiratory system^19^. In the case of alveolar macrophage, DEP exposure has induced the production of cytokines such as tumor necrosis factor-α (TNF-α), IL-1β, and IL-8 as well as cell apoptosis via mitochondrial dysfunction^20,21^. Additionally, portions of inhaled DEPs often translocate from the lungs to mediastinal lymph nodes and tend to activate dendritic cell (DC) function^22^. However, the effect that DEPs exert on other cells in the lymph nodes remains unclear.

Lymphatic endothelial cells (LECs) constitute the outer walls of lymph nodes and lymphatic vessels, and play a key role in immune regulation under both physiological and pathological conditions^23^. For example, DC translocation from the periphery to lymph nodes requires an interaction between the lymphatic vessel endothelial hyaluronan receptor-1 (LYVE-1) on LECs and DC-equipping hyaluronan^24^. In addition, LECs can bind various chemokines and consequently inhibit the recruitment of immune cells via chemokine scavenging receptor^25,26^. These factors have resulted in a recent increase in research concerning the inflammatory responses of LECs.

In this study, we used LECs that we had originally immortalized via transduction of temperature-sensitive SV40T in order to explore the effects of DEP exposure. We previously established an immortalized LEC cell line (iLEC) from mice and demonstrated that iLECs have the ability to form a tube in Matrigel and express LEC-marker genes such as LYVE-1 and Prospero-related homeobox1 (Prox1)^27^. Herein, we describe our comprehensive analysis of the changes in mRNA expression following DEP exposure and our investigation into the effect of DEPs on cell viability, cytokines production, and cellular response to stress.

## Materials and methods

An Annexin V-FITC Apoptosis Detection Kit, 4% paraformaldehyde (PFA), Hanks’ Balanced Salt Solution (HBSS), and phosphate-buffered saline without Ca^2+^ and Mg^2+^ (PBS) all were purchased from Nacalai Tesque (Kyoto, Japan). Accutase was purchased from Funakoshi (Tokyo, Japan). A Cell Counting Kit-8 (WST-8 assay) and a highly sensitive DCFH-DA ROS Assay Kit were obtained from Dojindo (Tokyo, Japan). A High-Capacity RNA-to-cDNA Kit and a Quant-iT PicoGreen dsDNA Assay Kit as well as dsDNA Reagents all were obtained from Thermo Fischer Scientific (Waltham, MA, U.S.A.). PrimeScript Reverse Transcriptase was purchased from TaKaRa-Bio (Shiga, Japan).

### Cell culture

Preparation of the immortalized mouse skin-derived lymphatic endothelial cell line (iLEC) was previously reported^27^. A collagen-coated solution was prepared by adding 1.5 mL of Type I-C (Nitta Gelatin, Osaka, Japan) to 43.5 mL of sterile water (pH 3.0) and passing the mixture through a 0.22 μm filter (Sartorius, Göttingen, Germany). A cell-culture dish was incubated with the collagen-coated solution for 30 min at 37 °C under a 5% CO_2_ atmosphere; the solution was discarded and the cell-culture dish was air-dried for 30 min in a sterile environment. Cells were cultured on the collagen-coated dishes in an Endothelial Cell Growth Medium MV Kit (PromoCell, Heidelberg, Germany), which was supplemented with 100 U/mL penicillin / 100 μg/mL streptomycin (Nacalai tesque) and 1 µg/mL Blasticidin S (FUJIFILM Wako Pure Chemical, Osaka, Japan). Cells were maintained at 33 °C under 5% CO_2_ conditions to activate the temperature-sensitive SV40T antigen and were sub-cultured from 1/3 to 1/5 every 2–3 days following detachment using Accutase (Funakoshi, Tokyo, Japan). The cells with less than 50 passages were used in the experiments. The numbers of cell seeding for the experiments were as follows: 2.4 × 10^5^ cells/well in 6-well plates, 6 × 10^4^ cells/well in 24-well plates, and 2 × 10^4^ cells/well in 96-well plates.

### Preparation of the solution containing DEP soluble and/or insoluble components

DEPs were collected as described previously^28^. Briefly, DEPs were collected at the National Institute for Environmental Studies (Tsukuba, Ibaraki, Japan). An 8 L diesel engine (J08C, Hino Motors, Tokyo) that was not fitted with post-exposure devices was powered under steady-state conditions (speed = 2000 rpm; engine torque = 0 Nm; diesel fuel = JIS No. 2) for 5 h. Particles were electrostatically (− 27 kVolts) collected at a distance of ≈10 m from the engine onto dichloromethane-washed gold discs at a flow rate of 20 L/min using a SSPM-100 sampler (Shimadzu, Kyoto, Japan).

DEPs were suspended in the culture medium; each concentration of DEP solution (0, 20, 50, 100, 200, 600 µg/mL) was prepared by stepwise dilution. In all experiments, the culture supernatant was removed, and the cells were exposed to each concentration of DEPs and incubated at 33 °C under 5% CO_2_ 24 h after seeding. An unexposed (without DEPs) control (Ctrl) was cultured in fresh medium. After incubation for the desired time, the cells were washed twice with PBS, and subjected to the desired manipulation.

To prepare soluble components of DEPs, 600 µg/mL of DEPs was suspended in PBS, and pipetted 10 times. After incubation for 10 min, the suspension was centrifuged (500 × *g*, 3 min) to precipitate the insoluble components of DEPs. The supernatant was collected, and diluted sixfold with medium to create a soluble component (equivalent to 100 μg/mL of DEPs). To prepare the core DEP suspension, the soluble compounds were washed out from the DEPs. Then, the precipitate was suspended in the culture medium and pipetted 10 times. Following incubation for 10 min at room temperature, the suspension was again centrifuged (500 × *g*, 3 min). This was repeated a total of 10 times. The final precipitate was weighed and then diluted with the culture medium at a concentration of 100 µg/mL.

### Evaluation of DEP-induced cell death

The iLECs in 24-well plates were exposed to DEP for either 6 or 24 h. In the ROS inhibition assay, DEPs were exposed to 5 mM N-Acetyl-L-cysteine (NAC). After removing the culture medium that contained the DEPs, a medium containing the 1 × WST-8 solution in the Cell Counting Kit-8 was added to each well, which was followed by incubation for 4 h. Absorbance at 450 nm (reference wavelength: 650 nm) was measured using the microplate reader Infinite 200 (TECAN, Männedorf, Switzerland). Survival rates were calculated by regarding the absorbance at 450 nm in DEP 0 µg/mL as 100%.

DEPs were internalized into the cells to examine the effect on cell viability; cellular uptake was blocked by incubating the DEPs under cool conditions. In these experiments, the cells were incubated in the presence of 10 mM HEPES for 2 h at 4 °C. After removal of the culture supernatant, the cells were exposed to 100 µg/mL of DEP and incubated at 4 °C for 6 h. A WST-8 assay was performed after incubating the iLECs with fresh culture medium at 33 °C for 1 h.

To determine which pathway was involved in DEP-induced cell death, the iLECs were incubated with DEPs in the presence of each inhibitor using 20 µM Z-DEVD-FMK (MEDICAL & BIOLOGICAL LABORATORIES) as an apoptosis inhibitor^29^, 150 µM Necrostatin 2 racemate (Nec-1 s, MedChemExpress) as a necroptosis inhibitor^30^, 2 µM Ferrostatin 1 (Fer-1, Abcam) as a ferroptosis inhibitor^31^, and 1 mM 3-Aminobenzamide (3-AB, Abcam) as a parthanatos inhibitor^32,33^. WST-8 assays were performed in the prescribed manner. Using the BZ-X71 (Keyence, Tokyo, Japan) with a 4 × objective lens; cell appearances were observed 6 h after addition of the DEPs.

### Transcriptome analysis using next-generation sequencing

The iLECs in 6-well plates were exposed to DEPs for 6 h at 20 and 100 μg/mL. Cells were detached using Accutase after washing with PBS, and then total RNA was extracted from the cells using the Maxwell RSC simplyRNA Tissue Kit and Maxwell RSC Instrument (Promega, Madison, WI, U.S.A). The extracted RNAs from 3 samples individually treated with each concentration of DEPs that were pooled in equal amounts for RNA-expression analysis. NGS analyses of the samples were outsourced to Veritas Genetics (Danvers, MA, U.S.A.). The obtained FASTQ files were annotated using STAR2.7.9a, and RNA expression was then quantified by processing the generated BAM files with RSEM1.3.3. The precise code during data analysis is shown in Supplemental methods. RNA expression was analyzed using iDEP.96 (http://bioinformatics.sdstate.edu/idep96/). Normalization was performed with Minimal counts per million in at least 3 libraries = 10; clustering by Heatmap and k-means methods and gene ontology analysis was applied to the top 3,000 genes using the standard deviation.

### Measurement of mRNA expression by quantitative reverse transcript polymerase chain reaction (qRT-PCR)

Cells in 6-well plates were exposed to DEPs for 6 h in indicated concentrations. To explore the effect that ROS inhibition exerted on DEP-induced toxicity, cells were exposed to a DEP suspension in the presence of 5 mM NAC. After washing with PBS, the cells were detached and total RNAs were extracted using the Maxwell RSC simplyRNA Tissue Kit (Promega). Total RNA was then reverse-transcribed and inverted using either the High-Capacity RNA-to-cDNA Kit or PrimeScript Reverse Transcriptase. Then, the obtained cDNA samples were subjected to qPCR using either the THUNDERBIRD SYBR qPCR Mix (TOYOBO, Osaka Japan) or the Vazyme Taq Pro Universal SYBR qPCR Master Mix (NIPPON Genetics, Tokyo, Japan). The sequences of the primers used in this study are listed in Supplemental Table 1.

### Measurement of DEP-induced ROS

The iLECs seeded onto 24-well plates were washed with HBSS and then incubated for 30 min with Highly Sensitive DCFH-DA Working solution in a ROS Assay Kit. After supernatant removal, the cells were exposed either to 100 µg/mL DEP or to 1 mM H_2_O_2_ for 1 h. After displacing the culture medium with a fresh medium, cells were observed using a confocal microscope LSM780 (ZEISS, Jena, German) with a 10 × objective lens. To quantitatively measure ROS production, the cells were exposed under the same conditions and analyzed via flow cytometry using NovoCyte (Agilent Technologies, Santa Clara, CA, U.S.A.).

### Raman imaging of intracellular DEPs

The iLECs in 6-well plates were exposed to 100 µg/mL of DEPs for 6 h. After washing, the cells were detached and centrifuged (500 *g*, 3 min). Cell pellets were suspended in a medium, dropped onto quartz glass substrates (DAICO MFG, Tokyo, Japan), and incubated for 2 h. After removal of the medium, the cells were incubated with a drop of 4% PFA for 10 min. After removing the PFA solution, the cell specimens were observed using a confocal Raman microscope via HR Evolution LabRAM (HORIBA, Kyoto Japan). Raman mapping was created using LabSpec6 software (HORIBA). The classical least squares (CLS) method was used to determine the component distribution after pre-processing with baseline correction, denoising, and SVD.

### Evaluation of apoptosis by flow cytometry

The iLECs in 6-well plates were incubated with 100 µg/mL of DEPs for 24 h. After removing the medium containing DEPs, the iLECs were detached using Accutase. The detached cells were stained with the Annexin V-FITC Apoptosis Detection Kit according to the manufacturer’s instructions. The stained cells were analyzed using NovoCyte. The cell fractions that were negative for propidium iodide (PI) and Annexin V were considered viable cell fractions.

### Quantification of IL-18 production by ELISA

The cells in 6-well plates were exposed to DEPs for 24 h, and then IL-18 concentration in the culture supernatant was evaluated via ELISA for IL-18 (MEDICAL & BIOLOGICAL LABORATORIES) according to the manufacturer’s instructions.

### Extracellular ATP (eATP) assay

The cells in 96-well plates were exposed to DEPs, and then treated with a RealTime-Glo Extracellular ATP Assay kit (Promega). Luminescence was measured continuously for 24 h using a plate reader iD5 (Molecular Devices, San Jose, CA, U.S.A.) under the following conditions: wavelength, all wavelengths; measurement time, 24 h; measurement interval, 1 min; Shake speed, low. As a positive control, 2 µM of the Staurosporine (FUJIFILM Wako Pure Chemical Corporation) group was used instead of DEPs.

### Extracellular double-stranded DNA (dsDNA) quantification via Picogreen assay

The cells in 6-well plates were exposed to either 100 µg/mL of DEPs or 2 µM of Staurosporine. At each time point, the dsDNA amount in the culture supernatant was measured using Quant-iT PicoGreen dsDNA Assay Kits with dsDNA Reagents according to the manufacturer’s protocol.

### Statistical analysis

For pair-wise comparisons, a Student’s t-test was performed. For a comparison of more than three groups, ANOVA followed by a Tukey’s HSD test was performed. If the P-value was lower than 0.05, the difference was regarded as statistically significant.

## Results

### The effect of DEP on LEC viability and transcriptome

**Figure 1:**
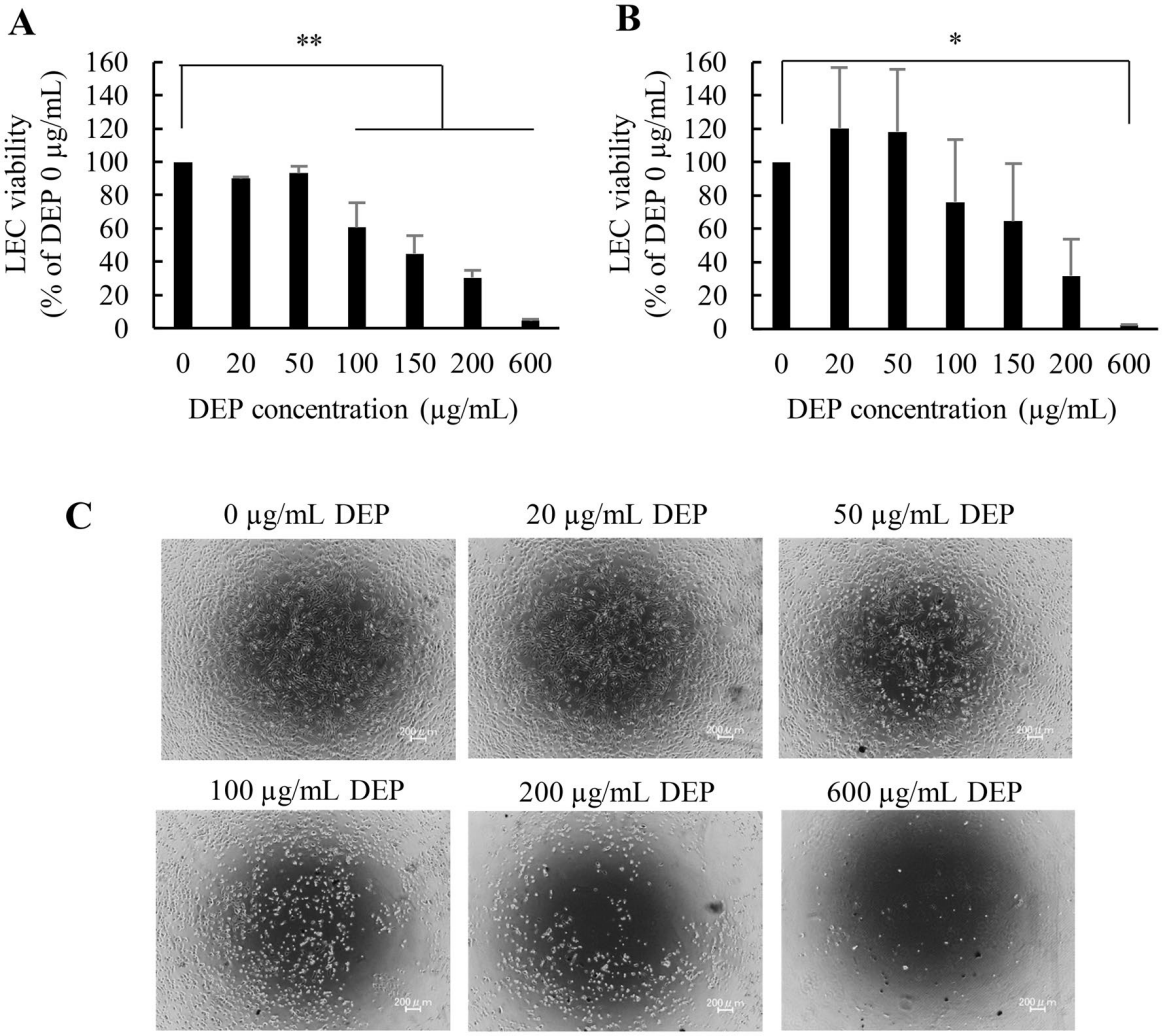
Cytotoxicity of iLECs following exposure to DEPs. (**A**), (**B**) Cell viability of LECs was evaluated following incubation with DEPs for (**A**) 6 h and B) 24 h. ANOVA was conducted, followed by Bonferroni testing (vs. DEP 0 μg/mL). *p < 0.05, **p < 0.01 (n = 3). Data represent the mean ± standard deviation (SD). (**C**) The morphology of iLECs was observed 6 h following exposure to DEPs.

Susceptibility to DEP exposure is variable among different types of cells. For example, DEPs exhibited a cell-killing effect in human lung epithelial cells (WI-38), in human bronchial epithelial cells at lower concentrations, and in human umbilical vein endothelial cells (< 100 μg/mL)0.17,34,35 DEPs showed marginal cytotoxicity to nasal fibroblasts and human macrophage-like cells (THP-1) even in high concentrations (> 500 μg/mL)0.36 To determine whether DEPs exhibited cytotoxic effects depending on concentration, the cell viability of iLECs was evaluated after either 6 or 24 h following exposure to DEPs at 0 ~ 600 μg/mL (Fig. 1A,B). As a result, cellular death appeared after 6 h. Moreover, exposure to > 100 μg/mL of DEPs induced significant cytotoxicity. At 600 μg/mL, almost all cells had died. A similar trend was also observed following 24 h of incubation. Long exposure to DEP didn’t significantly increase DEP-induced toxicity. As for morphology, dead cells morph into a round shape, which suggests an early stage of apoptosis (Fig. 1C). Of note, cell viability was increased at 20 and 50 μg/mL 24 h after the addition. Therefore, we then performed transcriptome analysis against the LECs treated with 20 and 100 μg/mL of DEP suspension.

**Figure 2:**
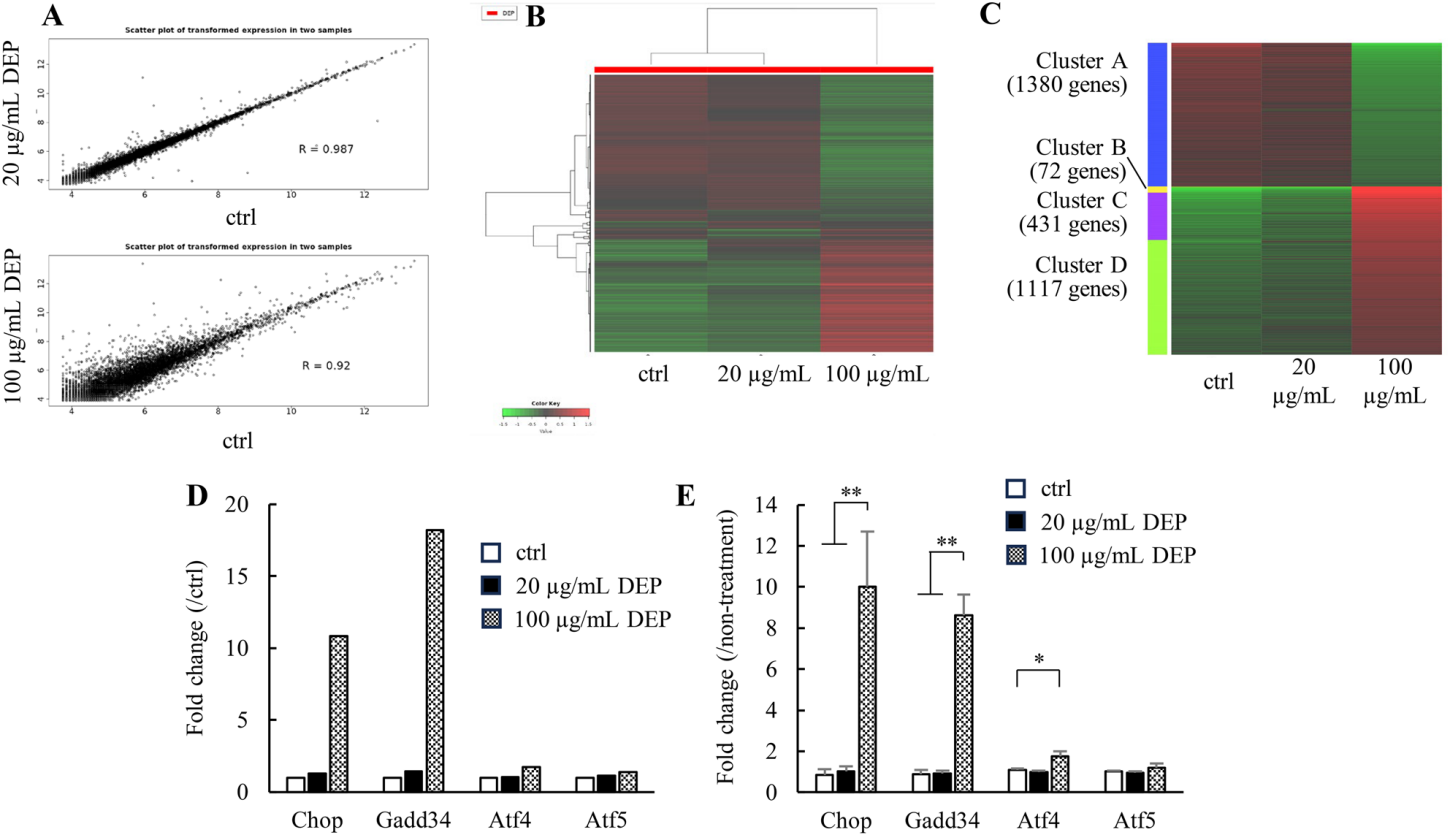
Transcriptome analysis of the gene expression fluctuation following 6 h of exposure to DEPs. (**A**) Scattered plot depicts 20 μg/mL vs. ctrl (upper panel) and 100 μg/mL vs. ctrl (lower panel). In the graphs, each dot refers to the normalized read counts of each gene in each group of measured RNA sequences. (**B**) Heatmap analysis was performed for the top 3000 genes where expression had changed the most. (**C**) Clustering analysis was conducted via the k-Means methods using 3000 genes where expression had changed the most, and k was set at 4. (**D**) ISR-related gene expression in transcriptome analysis. (**E**) The ISR-related gene expression was measured via qRT-PCR. ANOVA was conducted, followed by a Tukey’s HSD test (vs. DEP 0 μg/mL). *p < 0.05, **p < 0.01 (n = 3). Data are reported as the mean ± SD.

In previous literature, DEP exposure resulted in drastic changes in the signaling pathways such as in angiogenesis in various types of cells, in the nuclear Receptors Meta-pathway, and in adipogenesis in fibroblasts and epithelial cells^34^. To determine what cascades are affected by DEP exposure to iLECs, we performed comprehensive RNA expression analysis using NGS between the iLECs exposed to DEPs at 20 μg/mL (non-toxic) and 100 μg/mL (toxic) for 6 h. Exposure to a higher concentration (100 μg/mL) affected a larger number of genes by comparison with exposure to a lower concentration (20 μg/mL). Moreover, the extent of the variations in gene expressions was greater (Figs. 2A,B). The top 3,000 variable genes were clustered into 4 groups (Fig. 2C, Cluster A-D). Gene ontology (GO) analysis revealed that the genes related to cell death, to the cell stress response, to the metabolic system, and to transfer RNA (tRNA) synthesis and biosynthetic processes were found in clusters B and C wherein the genes were up-regulated only in 100 μg/mL of DEP (Supplemental Table 2). The overall cell responses to stress, referred to as the integrated stress response (ISR), are involved in these metabolic/catabolic processes^35^. We identified 4 ISR-related genes that were increased in the transcriptome data: *Chop* (*Ddit3*, DNA-damage inducible transcript), *Gadd34* (*Ppr1r15a*, protein phosphatase 1, regulatory subunit 15A), *Atf4* (*Creb2*, activating transcription factor 4), and *Atf5* (*Atfa*, activating transcription factor 5) **(**Fig. 2D**)**. Also, qRT-PCR clarified that DEP exposure elevated *Chop*, *Gadd34*, and *Atf4* (Fig. 2E).

### Insight into the mechanism of DEP-induced iLEC cytotoxicity

**Figure 3:**
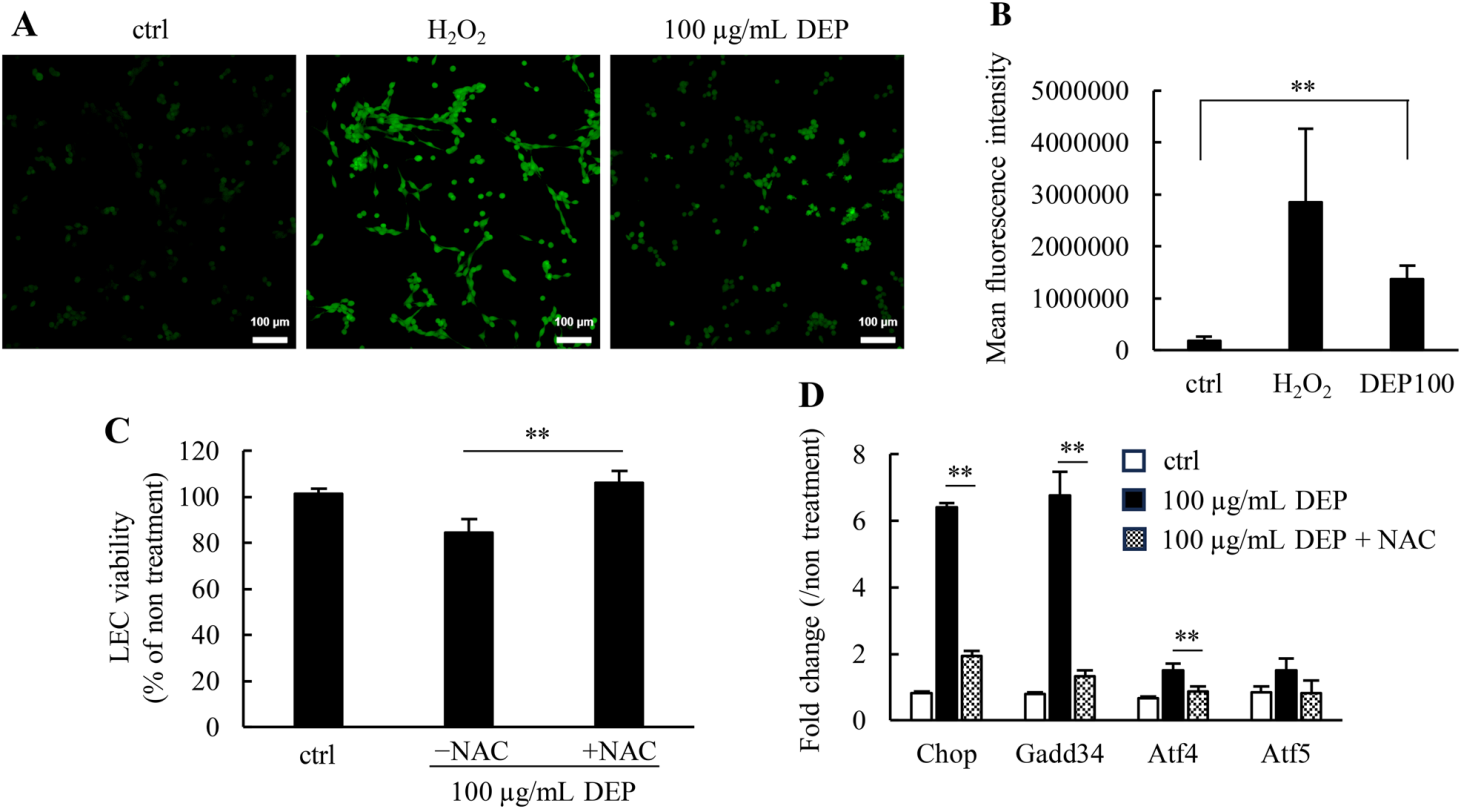
ISR-related genes up-regulation and cytotoxicity by intracellular ROS generated by exposure to DEPs. (**A**) Following DEP exposure, intracellular ROS was detected using a ROS Assay Kit. Green dots represent fluorescence colored by reacting ROS. Scale bars: 100 μm. (**B**) Cells were analyzed via flow cytometry following ROS exposure to DEPs. (**C**) DEP-induced cytotoxicity was measured in either the presence or absence of N-acetylcysteine (NAC). A Student’s t-test was performed to compare − NAC and + NAC. **p < 0.01 (n = 3). (**D**) The expressions of ISR-related genes were quantified via qRT-PCR in either the presence or absence of NAC. A Student’s t-test was performed to compare − NAC and + NAC. **p < 0.01 (n = 3). Data are reported as the mean ± SD.

To identify the molecular mechanism of the ISR of iLECs, we focused on the association between ISR genes and DEP-induced ROS, since ROS activated the ISR cascade via endoplasmic reticulum (ER) stress and oxidative stress^36,37^. To investigate ROS generation by DEPs, intracellular ROS was detected using ROS-reactive fluorescent dye (Fig. 3A,B). Cytotoxic concentrations of DEPs (100 μg/mL) produced substantial ROS, although the degree of ROS production was slightly lower than that of oxidant reagent hydrogen peroxide (H_2_O_2_). To confirm whether ROS are responsible for the cytotoxicity of DEPs, iLECs were exposed to DEPs in the presence of the anti-oxidant N-acetylcysteine (NAC). The cell viability decreased by DEPs was completely recovered by the addition of NAC (Fig. 3C). In addition, the ISR-related gene expressions activated by DEPs were partially cancelled in the presence of NAC (Fig. 3D).

**Figure 4:**
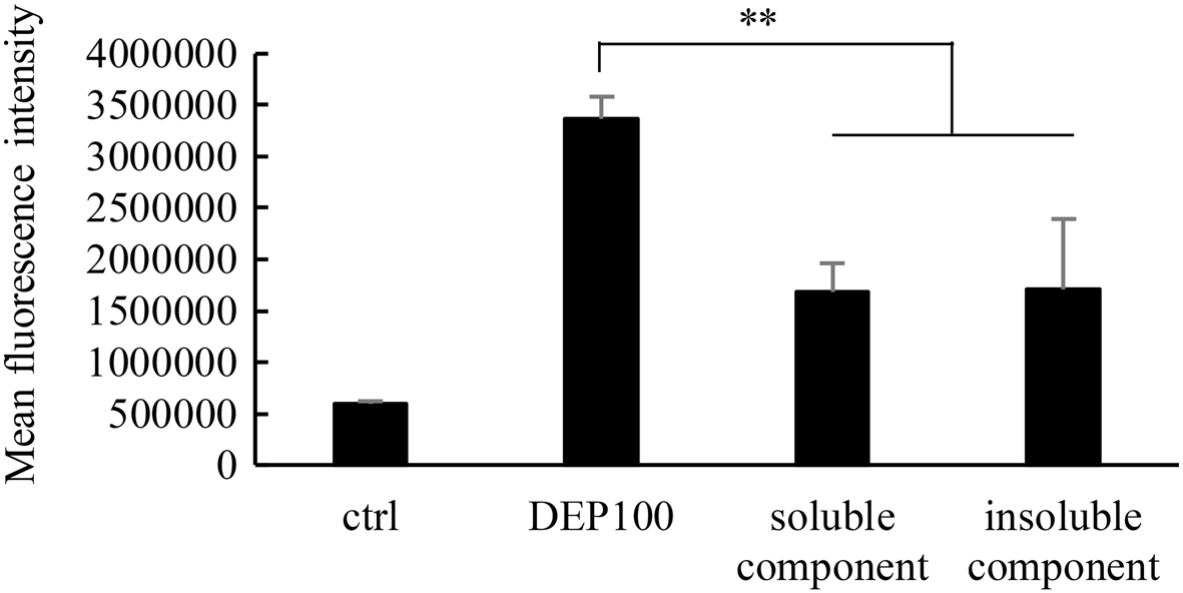
ROS generated by soluble/insoluble DEP components. Cell viabilities were measured following exposed to either soluble or insoluble components of DEPs for 6 h. The exposed concentrations of both soluble and insoluble components of DEPs were adjusted to an equivalent of 100 μg/mL of DEPs. ANOVA was conducted, followed by a Tukey’s HSD test (vs. DEP 100 μg/mL). *p < 0.05, **p < 0.01 (n = 3). Data are reported as the mean ± SD.

**Figure 5:**
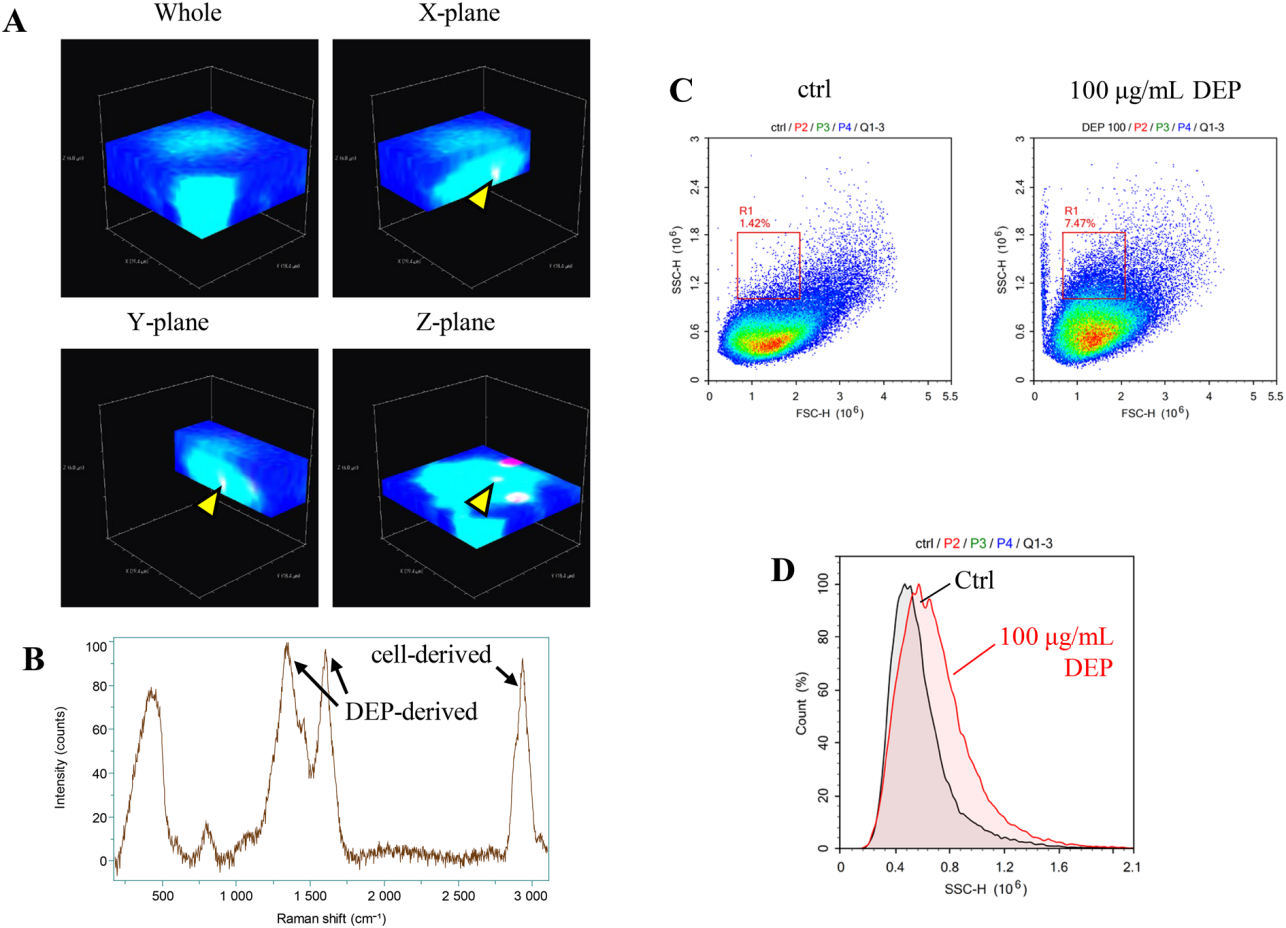
The internalization of DEPs into LECs. (**A**) A typical image of three-dimensional Raman imaging is shown. (**B**) The Raman shift spectrum is shown by the yellow arrowheads. (**C**) The dot plot represents iLECs exposed to 100 μg/mL of DEPs. The Y- and X-axes represent side scattering (SSC) and forward scattering (FSC), respectively. (**D**) A histogram of the SSC is depicted. Black and red lines indicate Ctrl and exposure to 100 μg/mL of DEPs, respectively.

To determine what components in DEPs were responsible for intracellular ROS induction, iLECs were separately exposed to both the water-soluble and water-insoluble components of DEPs (Fig. 4). As a result, each of the components generated intracellular ROS to a comparable extent (approximately half of that produced by whole DEPs). To observe the internalization of DEPs into iLECs, three-dimensional Raman imaging was performed as previously reported^38^. The Raman imaging revealed that DEPs were taken up by iLECs (Figs. 5A,B). This data were also supported by flow cytometry analysis. The side-scattering (SSC) in a portion of the iLECs was elevated by the exposure to DEPs by comparison with unexposed cells, as reported previously (Figs. 5C,D)^39,40^.

**Figure 6:**
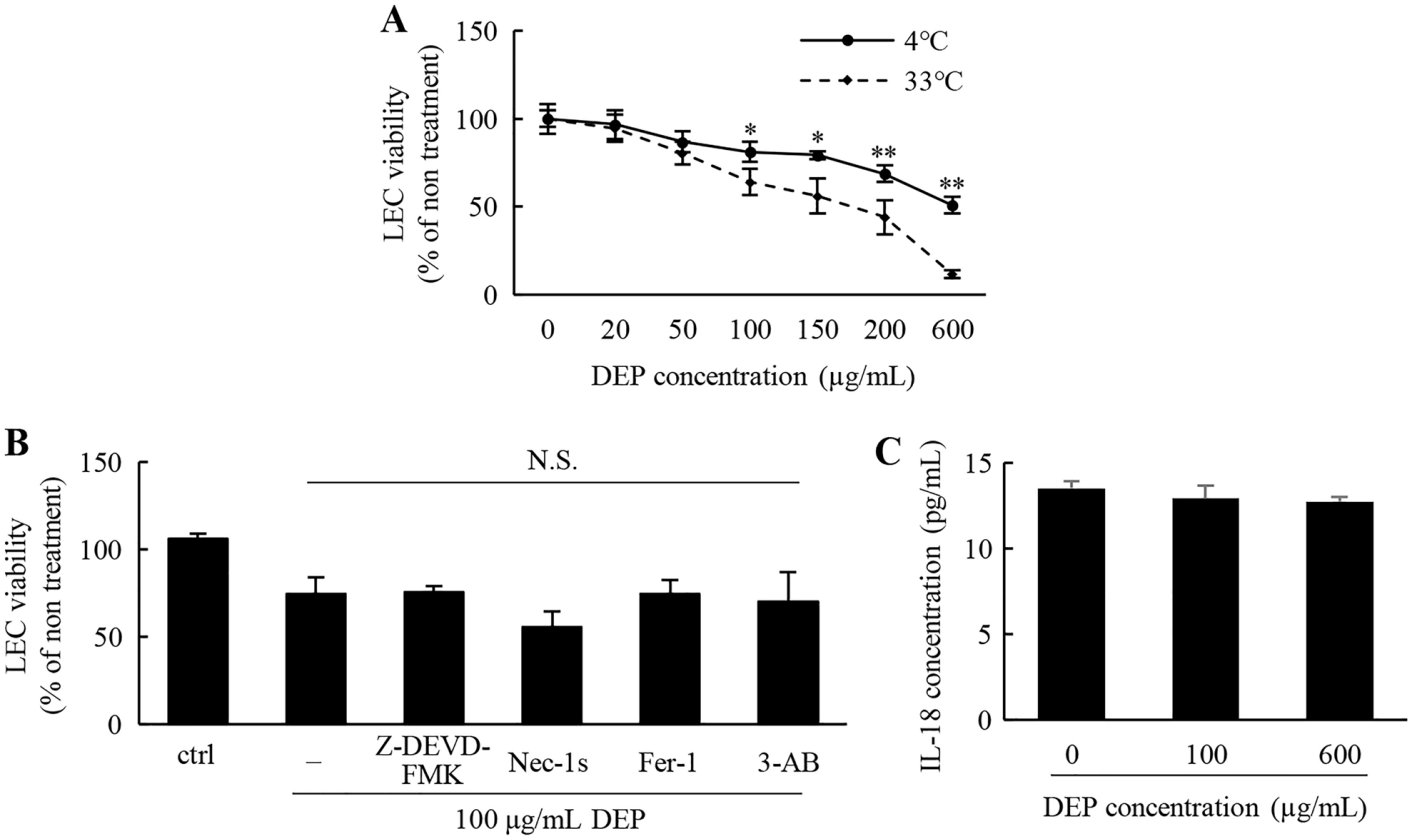
Effect of cell death inhibitors on cytotoxicity. (**A**) The DEP-induced cytotoxicity at 4 and 33 °C was evaluated at 6 h after exposure to DEPs in the indicated concentrations. (**B**) The inhibitory effect of the cell death inhibitors on the viability of LECs. The cells were exposed to 100 μg/mL of DEPs in the presence of a series of inhibitors for 6 h. (**C**) IL-18 concentration in the supernatant was measured after the cells were incubated with 100 and 600 μg/mL of DEPs for 6 h.

Finally, our investigation revealed the cell death cascade that is provoked by exposure to DEPs. During Annexin V-PI staining studies, late apoptotic/necrotic cells (Annexin V^+^/PI^+^) and necrotic cells (Annexin V^−^/PI^+^) appeared following exposure to high concentrations of DEPs (Figure S1). The DEP-induced cytotoxicity was significantly reduced at temperatures below 4 °C (Fig. 6A), which indicates that either the cell death and/or the DEP internalization is energy-dependent. This could suggest that the energy-dependent internalization of insoluble DEP components is necessary to exhibit toxicity. However, since the DEP-induced toxicity was not completely suppressed at 4 °C, the soluble factors that could enter LECs via passive diffusion would also be involved in cell death. These results are consistent with results showing that ROS produce both soluble and insoluble components to the same extent (Fig. 4). Additionally, we also detected a population of annexin V^−^/PI^+^, which are referred to as membrane-ruptured cells, which is presumably due to pyroptosis^41^. Moreover, necroptosis, ferroptosis, and parthanatos are known to be induced by environmental particulates^39,42^. Also, our NGS data suggest an elevation of the RNA expressions of these cascades (Suppelemtanl Table 3). To investigate the involvement of these cell death pathways, the cytotoxicity that is caused by DEPs was evaluated in the presence of the inhibitors of these cell death cascades. However, no inhibitors were able to relieve the DEP-induced cell death (Fig. 6B). There is no significant difference among DEP-treated groups. Although intracellular molecules such as IL-1β and IL-18 were released from the cytosol in pyroptosis^43,44^, DEP-treatment did not induce the leakage of IL-18 (Fig. 6C). In addition, there was no detection of the extracellular damage-associated molecular patterns (DAMPs) that are commonly regarded as pyroptosis markers such as double-stranded DNA (dsDNA) and ATPs (Figure S2).

## Discussion

The collective results show that ROS was responsible for the DEP-induced cytotoxicity in LECs. In other reports, ROS generated by DEP components has resulted in cell death in blood endothelial cells and in murine macrophage cells via apoptosis^45,46^. On the other hand, the DEP-induced deaths of iLECs were not cancelled by the apoptosis inhibitor Z-DEVD-FMK (Fig. 6B). Furthermore, PI/Annexin staining also supported the notion that apoptosis was caused by necrosis rather than by the DEP-induced cell death of iLECs (Figure S1). However, as inhibitors of necroptosis, ferroptosis and parthanatos showed only a marginal effect on cell viability, the cell death cascade that was induced by DEPs remains to be elucidated (Fig. 6B).

Gadd34, Atf4, and Chop are well-known genes involved in endoplasmic reticulum (ER) stress, which is typically activated via responses to incorrectly folded proteins, called unfolded protein response (UPR)^47^. During UPR, phosphorylated eIF2α facilitates ATF4 expression, and subsequently ATF4 translocates to the nuclei, and enhances the genes related to UPR-mediated functions such as the inducement of CHOP. CHOP begins the mediation of cell apoptosis in response to cell stress-inducible apoptosis^48^. In this context, Gadd34 supports the de-phosphorylation of eIF2α as negative feedback in the process of apoptosis as a result of UPR. Although the relationship between ER stress and ROS is not fully understood, a previous study revealed that H_2_O_2_ increased CHOP expression in rat neurocytes^49^. It could be the case that oxidative stress can induce ER stress in LECs as well.

Since ISR-related genes were elevated by exposure to DEPs, we hypothesized that the ISR may be related to the cell death. However, ISRIB, an inhibitor of the ISR pathway, in turn, showed no effect on the DEP-induced cytotoxicity. This indicates that the ISR and cell death were apparently independently provoked by ROS (Figure S3). Taken together, both the soluble and the insoluble components of DEPs would generate ROS in the iLECs and the subsequent ROS-induced cell death, which likely is unrelated to the ISR.

While the mechanism for cell death remains to be elucidated, the internalization of DEPs into LECs is likely to be essential for ROS generation and for DEP-induced cell death since insoluble DEP components (core DEPs), as well as soluble components of DEPs likely generate the intracellular ROS (Figure 4), and, in fact, Raman imaging and flow cytometric analysis suggest the internalization of DEPs (Figure 5). Although previous studies revealed that soluble components such as PAH and nitro-PAH produce ROS through intracellular metabolism of their organic compounds, how insoluble DEP components generate ROS remains unclear^50^. Since a previous study suggested that the carbon core *per se* altered the reaction of immune cells such as whole DEPs, the insoluble DEP core has the ability to affect cellular function^51^.

With respect to stromal cells in the lymph nodes, LECs play multifaceted roles in immune regulation, particularly in suppression^52^. For example, LEC expresses CD73, which catalyzes the synthesis of extracellular adenosine, which is an immunosuppressive metabolite^53,54^. Adenosine synthesized by CD73 in LECs inhibited both cell proliferation and the up-regulation of immune-activating proteins such as the CD40 and CD80 of DC. Given that LEC function was disrupted by DEP-induced cytotoxicity, inflammation could be aggravated synergistically by the direct immune-stimulative effect of DEPs on immune cells^55,56^.

A limitation of this study is a lack of information about the differences in the LEC features between mice and humans. Recent studies have revealed many differences in the expressions of marker proteins such as LYVE1, programmed death-1 ligands, and Macrophage receptors with collagenous structures, although a huge amount of functional and morphological aspects were found to be highly conserved among all species^23^. Since it is possible that the effect that DEP-exposure exerts on murine LECs might not be identical to humans, further study should be considered for a clarification of the DEP-induced toxicity to human LECs.

In this study, we did not demonstrate NGS analysis of DEP-treated cells in the presence of NAC. Thus, the contribution of the ROS generated by DEP treatment to the changes of individual gene expression remains unclear. To understand the multiple effect of DEP on LECs, further studies should be conducted.

## Conclusions

In this study, we investigated the effect of DEP exposure on gene expression profiles and cytotoxicity in LECs. Comprehensive RNA-seq revealed that exposure to DEPs up-regulates the cell death cascades and ISR-related genes. Antioxidants relieved the cytotoxic effect, which revealed that DEP-induced cytotoxicity was caused by ROS, which was produced by both water-soluble and insoluble components of DEPs. Further analysis on the mechanisms of cell death and intracellular signaling is essential to better understand how DEPs that migrate to the lymphatic system affect in vivo immune responses.

## Data and code availability

These RNA sequencing data are stored in the DNA Data Bank of Japan (DDBJ) Sequence Read Archive (SRA) database (Accession No. DRA017533). The code for analysis was mentioned in Supplemental Information.

### Supplementary Information

Supplementary Information.
