# Supplementary material for: The stress-responsive cytotoxic effect of diesel exhaust particles on lymphatic endothelial cells
Source: Sci Rep. 2024 May 7;14:10503. doi: 10.1038/s41598-024-61255-4 (PMC11076499; doi:10.1038/s41598-024-61255-4)
Supplement: Supplementary file 1 — Supplementary Information. [file 41598_2024_61255_MOESM1_ESM.docx]

**Supplemental Table 1 The sequences of primer for qRT-PCR**

| Primers | Sequence (3’ to 5’) |
| --- | --- |
| Gapdh Forward | AGCAAGGACACTGAGCAAG |
| Gapdh Reverse | TAGGCCCCTCCTGTTATTATG |
| Chop Forward | CCTAGCTTGGCTGACAGAGG |
| Chop Reverse | CTGCTCCTTCTCCTTCATGC |
| Gadd34 Forward | TACCCCTGTCTCTGGTAACCT |
| Gadd34 Reverse | TGGCTTTGCATTGTACTCATCA |
| Atf4 Forward | GCCGGTTTAAGTTGTGTGCT |
| Atf4 Reverse | CTGGATTCGAGGAATGTGCT |
| Atf5 Forward | CTACCCCTCCATTCCACTTTCC |
| Atf5 Reverse | TTCTTGACTGGCTTCTCACTTGTG |

**Supplemental Table 2. The clusterization of Gene Ontology with a significant difference**

| Cluster | Adj. P-value | Genes | Pathways |
| --- | --- | --- | --- |
| A | 9.72E-09 | 137 | Mitotic cell cycle |
| A | 1.36E-08 | 70 | Chromosome segregation |
| A | 2.37E-08 | 117 | Mitotic cell cycle process |
| A | 7.94E-08 | 214 | Cell cycle |
| A | 5.05E-07 | 405 | Organelle organization |
| A | 5.25E-07 | 156 | Cell cycle process |
| A | 5.25E-07 | 81 | Organelle fission |
| A | 5.26E-07 | 58 | Mitotic nuclear division |
| A | 5.26E-07 | 74 | Nuclear division |
| A | 5.26E-07 | 99 | Cell division |
| A | 1.35E-06 | 98 | Regulation of cell cycle process |
| A | 2.49E-06 | 54 | Nuclear chromosome segregation |
| A | 5.41E-06 | 40 | Mitotic sister chromatid segregation |
| A | 1.49E-05 | 43 | Sister chromatid segregation |
| A | 2.01E-05 | 147 | Regulation of organelle organization |
| B | 2.18E-12 | 33 | Apoptotic process |
| B | 2.18E-12 | 34 | Cell death |
| B | 2.18E-12 | 33 | Programmed cell death |
| B | 1.21E-10 | 29 | Regulation of cell death |
| B | 3.50E-10 | 27 | Regulation of apoptotic process |
| B | 3.75E-10 | 31 | Cellular response to stress |
| B | 4.25E-10 | 27 | Regulation of programmed cell death |
| B | 2.17E-09 | 38 | Response to stress |
| B | 1.23E-08 | 20 | Negative regulation of apoptotic process |
| B | 1.23E-08 | 31 | Negative regulation of nitrogen compound metabolic process |
| B | 1.59E-08 | 20 | Negative regulation of programmed cell death |
| B | 3.70E-08 | 13 | Intrinsic apoptotic signaling pathway |
| B | 4.44E-08 | 20 | Negative regulation of cellular protein metabolic process |
| B | 5.37E-08 | 17 | Regulation of proteolysis |
| B | 8.69E-08 | 42 | Negative regulation of cellular process |
| C | 0.000852 | 133 | Positive regulation of metabolic process |
| C | 0.001205 | 161 | Cellular macromolecule biosynthetic process |
| C | 0.001405 | 161 | Macromolecule biosynthetic process |
| C | 0.002419 | 121 | Positive regulation of macromolecule metabolic process |
| C | 0.002595 | 9 | TRNA aminoacylation for protein translation |
| C | 0.002595 | 20 | Cellular amino acid metabolic process |
| C | 0.002959 | 106 | Response to organic substance |
| C | 0.003209 | 149 | Regulation of gene expression |
| C | 0.003209 | 9 | Amino acid activation |
| C | 0.003209 | 9 | TRNA aminoacylation |
| C | 0.003209 | 157 | Negative regulation of cellular process |
| C | 0.004794 | 49 | Protein catabolic process |
| C | 0.00555 | 127 | Regulation of cellular macromolecule biosynthetic process |
| C | 0.005648 | 60 | Macromolecule catabolic process |
| C | 0.005648 | 134 | Regulation of biosynthetic process |
| D | 3.12E-12 | 154 | Cellular macromolecule catabolic process |
| D | 3.12E-12 | 432 | Cellular protein metabolic process |
| D | 4.16E-12 | 169 | Macromolecule catabolic process |
| D | 7.22E-12 | 227 | Cellular catabolic process |
| D | 8.68E-11 | 118 | Cellular protein catabolic process |
| D | 1.33E-10 | 131 | Protein catabolic process |
| D | 1.51E-10 | 430 | Nucleic acid metabolic process |
| D | 1.76E-10 | 131 | Protein modification by small protein conjugation or removal |
| D | 2.29E-10 | 242 | Catabolic process |
| D | 3.42E-10 | 309 | Positive regulation of macromolecule metabolic process |
| D | 4.15E-10 | 97 | Ubiquitin-dependent protein catabolic process |
| D | 6.63E-10 | 151 | Organonitrogen compound catabolic process |
| D | 6.63E-10 | 186 | Positive regulation of RNA metabolic process |
| D | 7.61E-10 | 108 | Proteolysis involved in cellular protein catabolic process |
| D | 1.14E-09 | 208 | Organic substance catabolic process |

**Supplemental Table 3. The expression of the genes involved in cell death cascades**

|  | Gene | Ctrl (TPM) | 20 μg/ mL DEP (TPM) | 100 μg/mL DEP (TPM) |
| --- | --- | --- | --- | --- |
| Apoptosis | Mdm2 | 110.88 | 122.36 | 174.61 |
|  | Casp3 | 70.65 | 65.39 | 82.43 |
|  | Casp7 | 8.05 | 6.89 | 5.75 |
|  | Casp8 | 28.6 | 26.07 | 16.76 |
|  | Casp9 | 9.87 | 9.2 | 6.36 |
|  | Casp12 | 16.92 | 15.99 | 12.09 |
|  | Bax | 124.61 | 135.94 | 130.98 |
|  | Bad | 47.4 | 44.24 | 22.82 |
|  | Cycs | 7.53 | 8.2 | 6.21 |
| Pyroptosis | Nlrp3 | 0.01 | 0 | 0.16 |
|  | Nlrc4 | 0.01 | 0.1 | 0.03 |
|  | Casp1 | 0.79 | 0.77 | 0.92 |
|  | Casp4 | 1.92 | 1.88 | 3.9 |
|  | Gsdmd | 6.52 | 9.13 | 4.63 |
|  | Il18 | 2.87 | 4.53 | 3.18 |
| Necroptosis | Ripk1 | 23.45 | 25.09 | 31.71 |
|  | Ripk3 | 17.52 | 17.43 | 16.84 |
|  | Mlkl | 50.55 | 59.94 | 45.22 |
| Ferroptosis | Slc7a11 | 5.92 | 100.26 | 222.25 |
|  | Slc3a2 | 214.03 | 337.29 | 955.51 |
|  | Ftl1 | 2078.72 | 3276.71 | 6004.34 |
|  | Fth1 | 3048.08 | 4758.05 | 5443.23 |
|  | Gpx4 | 471.46 | 466.96 | 441.73 |
| Integrated stress response | Ddit3 (Chop) | 76.17 | 95.82 | 824.22 |
|  | Ppp1r15a (Gadd34) | 18.66 | 27.1 | 339.23 |
|  | Atf4 | 375.93 | 390.25 | 648.2 |
|  | Atf5 | 108.6 | 123.91 | 147.84 |

*TPM: transcripts per million


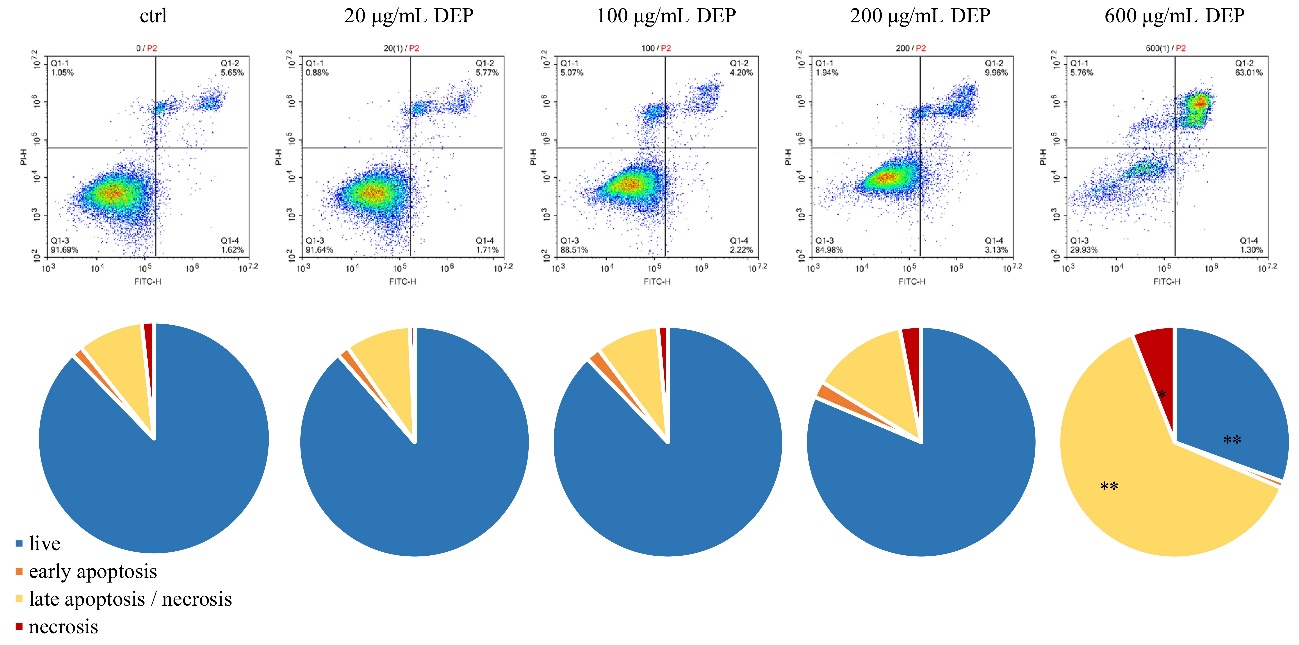


**Supplemental Figure 1 PI/Annexin V staining of DEP-treated LEC.** LEC was exposed to DEP at indicated concentrations for 6 h. Then, cells were stained by PI/Annexin V.


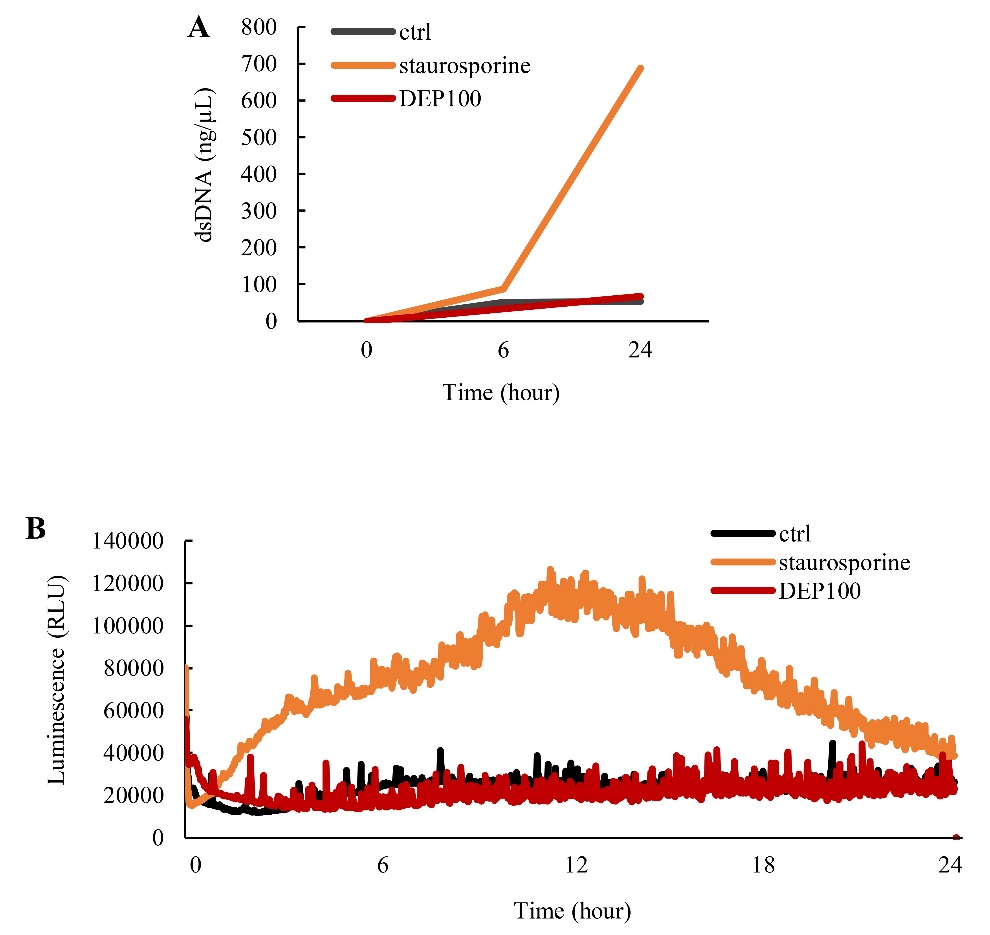


**Supplemental Figure 2 The evaluation of damage-associated pattern molecules (DAMPs).** A) The concentration of dsDNA in the supernatant was measured 6 h after the addition of DEP. B) The extracellular ATP was measured in the presence of 100 μg/mL DEP.


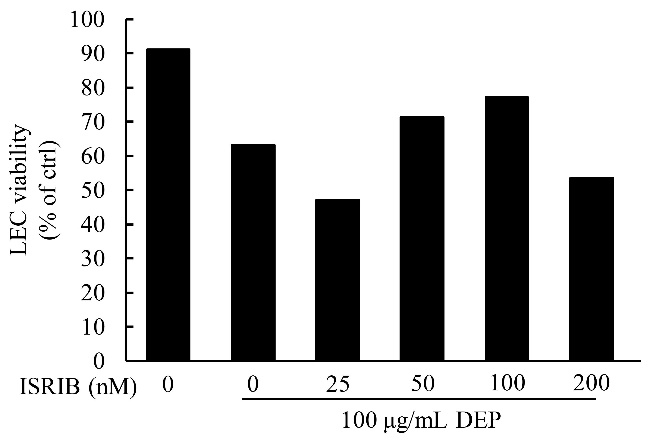


**Supplemental Figure 3 The inhibitory effect of ISR inhibitor on the cytotoxicity of DEP.** LEC was incubated with 100 μg/mL DEP in the presence of 0 – 200 nM ISR inhibitor, ISRIB.

**Supplemental methods**

**Determination of RNA expressions from NGS data**

NGS data was processed in reference to the book (ISBN: 478090983X). To map FASTQ data to reference genome, reference data (Mus_musculus_GRCm39.dna.103.primary_assembly.fa.gz and annotation file (Mus_musculus.GRCm39.103.gtf.gz) were downloaded from Ensemble.org. Then the gene name was linked to the gene ID with R 4.1.2 software. Through BiocManager, biomaRt and dplyr were installed into R in prior and then the below code was executed.

*> mart <- biomaRt::useMart(biomart = “ENSEMBL_MART_ENSEMBL”, dataset =*

*“mmusculus_gene_ensembl”, host = ‘ensembl.org’)*

*> t2g <- biomaRt::getBM(attributes = c(“ensembl_transcript_id”, “ensemble_gene_id”,*

*“external_gene_name”), mart = mart*

*> t2g <- dplyr::rename(t2g, target_id = ensembe_transcript_id, ens_gene =*

*Ensemble_gene_id, ext_gene = external_gene_name)*

*> t2g[t2g[,3] == “”,”ext_gene”] <- “NA”*

*> write.table(t2g, “target2gene.txt, sep=” \t”,quote=F,row.names=F)*

For mapping RNA-seq data to reference genome data, STAR2.7.9a from <https://github.com/alexdobin/STAR/archive> and then index file was created and subsequently fastq data were mapped to reference genomes by executing the below code for all fastq files in the Terminal.

*$ ~/Documents/expression/tools/STAR-2.7.9a/bin/MacOSX_x86_64/STAR --runMode*

*genomeGenerate –genomeDir ~/Documents/expression/ref/STAR_reference*

*--genomeFastaFiles ~/Documents/expression/ref/Mus_musculus.GRCm39.dna.primary_*

*assembly.fa –sjdbGTFfile ~/Documents/expression/ref/Mus_musculus.GRCm39.103.gtf*

*$ ~/Documents/expression/tools/STAR-2.7.9a/bin/MacOSX_x86_64/STAR --runMode*

*alignReads --genomeDir ../ref/STAR_reference --readFilesCommand gunzip -c*

*--readFilesIn ../seq/{sample_name}_1.fastq.gz ../seq/{sample_name}_2.fastq.gz*

*--outSAMtype BAM SortedByCoordinate --runThreadN 4 --outFilePrefix {sample_name}*

*--quantMode TanscriptomeSAM*

Then RNA expression was evaluated through the BAM files generated by STAR2.7.9a using RSEM-1.3.3 from “deweylab.github.io/RSEM/”. Index files for RSEM-1.3.3 was prepared and then each RNA expression levels were determined as follows:

*$ ../tools/RSEM-1.3.3/bin/rsem-prepare-reference -num-threads 4 --gtf*

*Mus_musculus.GRCm39.103.gtf Mus_musculus.GRCm39.dna.primary_assembly. fa*

*RSEM_reference/RSEM_reference*

*$ cd ~/Documents/expression/STAR*

*$ ../tools.RSEM-1.3.3/rsem-calculate-expression --num-threads 4 --paired-end --bam*

*{sample_name}Aligned.toTranscriptome.out.bam ../re/RSEM_reference/RSEM_reference*

Finally, the generated “{sample_name}.genes.results” files were analyzed by browser based-analysis software, iDEP.
